# Improving broad-coverage medical entity linking with semantic type prediction and large-scale datasets

**DOI:** 10.1016/j.jbi.2021.103880

**Published:** 2021-08-12

**Authors:** Shikhar Vashishth, Denis Newman-Griffis, Rishabh Joshi, Ritam Dutt, Carolyn P. Rosé

**Affiliations:** a Carnegie Mellon University, 5000 Forbes Ave, Pittsburgh, PA, USA; b University of Pittsburgh, 5607 Baum Blvd, Pittsburgh, PA, USA

**Keywords:** Natural language processing, Information extraction, Medical concept normalization, Medical entity linking, Distant supervision, Entity typing

## Abstract

**Objectives:**

Biomedical natural language processing tools are increasingly being applied for broad-coverage information extraction—extracting medical information of all types in a scientific document or a clinical note. In such broad-coverage settings, linking mentions of medical concepts to standardized vocabularies requires choosing the best candidate concepts from large inventories covering dozens of types. This study presents a novel semantic type prediction module for biomedical NLP pipelines and two automatically-constructed, large-scale datasets with broad coverage of semantic types.

**Methods:**

We experiment with five off-the-shelf biomedical NLP toolkits on four benchmark datasets for medical information extraction from scientific literature and clinical notes. All toolkits adopt a staged approach of mention detection followed by two stages of medical entity linking: (1) generating a list of candidate concepts, and (2) picking the best concept among them. We introduce a *semantic type prediction* module to alleviate the problem of overgeneration of candidate concepts by filtering out irrelevant candidate concepts based on the predicted semantic type of a mention. We present MedType, a fully modular semantic type prediction model which we integrate into the existing NLP toolkits. To address the dearth of broad-coverage training data for medical information extraction, we further present WikiMed and PubMedDS, two large-scale datasets for medical entity linking.

**Results:**

Semantic type filtering improves medical entity linking performance across all toolkits and datasets, often by several percentage points of F-1. Further, pretraining MedType on our novel datasets achieves state-of-the-art performance for semantic type prediction in biomedical text.

**Conclusions:**

Semantic type prediction is a key part of building accurate NLP pipelines for broad-coverage information extraction from biomedical text. We make our source code and novel datasets publicly available to foster reproducible research.

## 1. Introduction

Biomedical natural language processing (NLP) tools are increasingly being applied for a wide variety of purposes, from clinical research [1] to quality improvement [2]. One of the key ways in which these tools are used is for broad-coverage information extraction: identifying all of the biomedical concepts, of all types, that are mentioned in a given document. Several well-known biomedical NLP tools have been developed as standalone software packages and are regularly used for broad-coverage extraction in non-NLP research: for example, cTAKES [3] has been explored for ischemic stroke classification [4] and studying infection risk [5]; and MetaMap [6] is frequently used in pharmacovigilance [7] and has even been adapted to health outcomes study in social media [8].

One of the central challenges in broad-coverage information extraction is the diversity of concepts in the standardized vocabularies that form the backbone of biomedical text analysis [9]. For example, the Unified Medical Language System, or UMLS [10], Metathesaurus contains over 3.5 million unique concepts belonging to 127 different semantic types.^1^ While much of the prior research on biomedical NLP methods has focused on restricted subsets of concepts, such as diseases and disorders or genes and proteins [11], general-purpose tools built for arbitrary use must deal with the full breadth of concept types in reference vocabularies.

In this study, we propose *semantic type prediction* as a key component of general-purpose biomedical NLP pipelines. Existing pipelines generally take a multi-stage approach to information extraction that is a natural fit for integrating semantic type prediction. The first stage is *mention detection* (also referred to as named entity recognition, or NER), which involves identifying textual mentions corresponding to different medical concepts of interest. The second stage is *medical entity linking* (also referred to as medical concept normalization, or MCN [12]), which can broadly be broken into two phases of *candidate generation*—identifying a set of standardized concepts a specific mention may refer to—and *disambiguation*—picking the best candidate concept for the observed mention based on the context (includes both word and phrase sense disambiguation, or WSD).

Compared to mention detection and disambiguation, candidate generation is an under-studied component of medical information extraction. Prior methods have historically relied on dictionary lookup and string matching [6,3] for both NER and candidate generation, yielding high precision but incomplete coverage [13,14]. Recent neural methods have taken an opposite approach to the problem by using entire concept inventories as candidates, providing complete coverage at the cost of large candidate set sizes [15–18]. However, this approach rapidly becomes intractable when generalizing to wider-coverage vocabularies. Thus, robust strategies to reduce *overgeneration* of candidates are required to leverage the high coverage afforded by neural approaches for a broad-coverage setting.

In addition to cataloguing known surface forms for medical concepts, the UMLS Metathesaurus also assigns each concept one or more semantic types; these types present a significant and under-utilized resource for balancing coverage with candidate set size in medical entity linking. In addition to limiting the set of candidate concepts in full-inventory approaches, semantic type information can reduce problems of ambiguity in text [19–21]. For example, the string *cold* can refer to *common cold* (disease), *cold temperature* (natural phenomena), or *cold brand* (pharmacologic substance) in different contexts. Semantic type prediction can thus inform both full-inventory and dictionary-based approaches to medical entity linking.

Identifying the semantic type of mentions has previously been shown to improve entity linking performance in Wikipedia [22]. However, this idea has not yet been systematically explored for medical entity linking, in part due to the dearth of annotated training data for the task. Curation of new biomedical text datasets faces significant barriers in the difficulty and cost of finding expert annotators [23] as well as the confidentiality and privacy issues inherent in sharing medical data [24]. These problems are only compounded in the broad-coverage setting, where data must be sufficiently diverse to represent all the kinds of information users of NLP systems may be interested in.

**Fig. 1.**
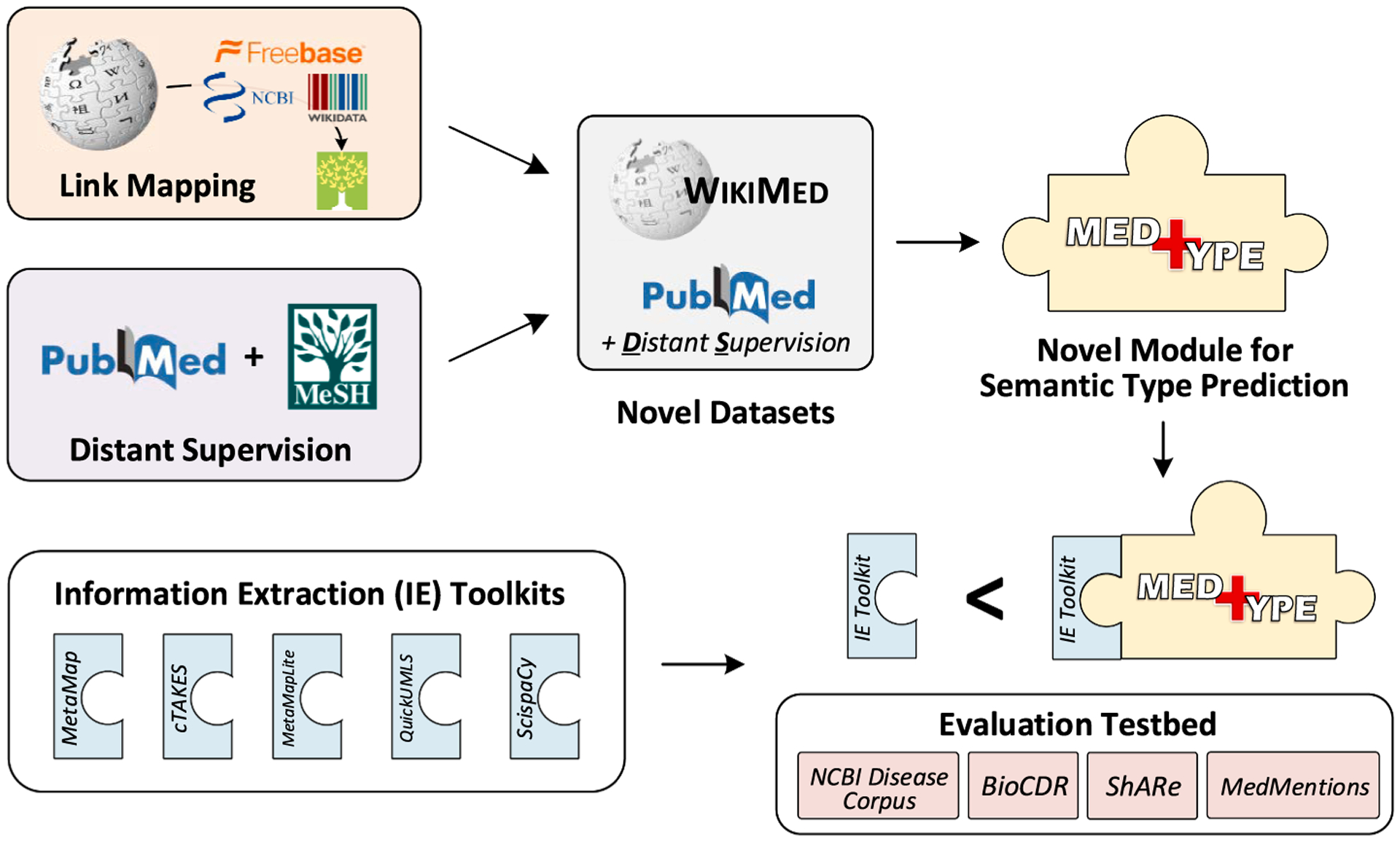
Overview of article contributions. We present MedType, a novel, modular system for biomedical semantic type prediction, together with WikiMed and PubMedDS, two large-scale, automatically created datasets for medical concept normalization that we use to pretrain MedType. We show that integrating MedType with five commonly used packages for biomedical information extraction improves performance across the board on four benchmark datasets.

This article presents two significant innovations, illustrated in Fig. 1: (1) a fully modular approach to alleviating candidate set overgeneration in medical entity linking via semantic type prediction, and (2) two large-scale datasets for medical entity linking research that are freely shareable. We make the following contributions:

- We present MedType, a deep learning-based modular system for semantic type prediction, and incorporate it into five off-the-shelf toolkits for medical entity linking. We demonstrate that semantic type prediction consistently improves entity linking performance across several benchmark datasets.
- To address the dearth of annotated training data for medical entity linking, we present WikiMed and PubMedDS, two automatically-created, large-scale datasets which can serve as a useful resource for medical entity linking research. Our work also demonstrates that pre-training MedType on our proposed datasets achieves state-of-the-art performance on the semantic type prediction task.
- We show that type-based filtering significantly reduces the number of candidates for disambiguation, enabling further improvements in the final step of medical entity linking.

MedType’s source code and the WikiMed and PubMedDS datasets proposed in this paper have been made publicly available at http://github.com/svjan5/medtype.

The remainder of this article is organized as follows. Section 2 highlights related work in the foundational NLP methods and medical NLP literature leading to our work on semantic type filtering. Section 3 introduces semantic type filtering as a component of the medical information extraction pipeline, and presents MedType, our state-of-the-art model for biomedical semantic type prediction. Section 4 describes our two novel, large-scale corpora, including quality assessments of each corpus. Section 5 describes our experimental protocol, and Section 6 presents the results of our analysis. Finally, Section 7 discusses implications of our findings for research on broad-coverage information extraction, and Section 8 concludes the paper.

## 2. Related work

Information extraction is a well-studied task in NLP, and approaches often diverge between the foundational methodologies literature, which typically utilizes news wire or web text, and the medical NLP literature, which reflects adaptations to the unique characteristics of biomedical text and knowledge (e.g., specialized language, rich typologies, etc.). In this paper, we combine recent insights from foundational methods with the rich expert resources that are central to biomedical information extraction.

Much of the research in the foundational methods literature focuses on extracting information about real-world entities and concepts (people, places, organizations, products, etc.), drawing on knowledge sources such as Freebase and Wikipedia. In addition to jointly modeling NER and entity linking as interdependent tasks [25,26], many studies leverage the rich semantics of the target knowledge base to improve linking performance [27,28]. Knowledge bases often group entities into semantic types, which inform several downstream NLP tasks such as co-reference resolution [29], relation extraction [30], question answering [31], and language modeling [32]. Recent studies have shown that fine-grained entity type prediction improves entity linking in Wikipedia text [33,22], indicating a clear potential for type prediction as a standard component of entity linking pipelines.

In the biomedical domain, the role of entity type prediction in selecting suitable candidates for medical concept mentions was recognized in some of the earliest rule-based medical information extraction tools [34]. However, type prediction is typically deeply embedded in rule-based NLP tools, hampering generalizability, and discourages their use in deep learning systems. [35] utilized neural language modeling frameworks to identify the semantic type of a mention in a medical text, but did not apply their predictions downstream; in contrast, [36] utilized approximate dictionary matching heuristics with specialized neural language models to improve both medical entity typing and entity linking in biomedical literature. However, these works have not explored the efficacy of incorporating the type information within the entity linking task itself. Zhu et al. model mention and entity types as latent variable and jointly optimize type learning and entity disambiguation. Our work alleviates the overgeneration problem produced by both rule-based [14] and deep learning systems in practical broad-coverage settings, by using the predicted semantic type to prune irrelevant candidates. We do so in a modular fashion, making it easy to incorporate in any entity linking architecture.

## 3. Semantic type prediction with MedType

**Fig. 2.**
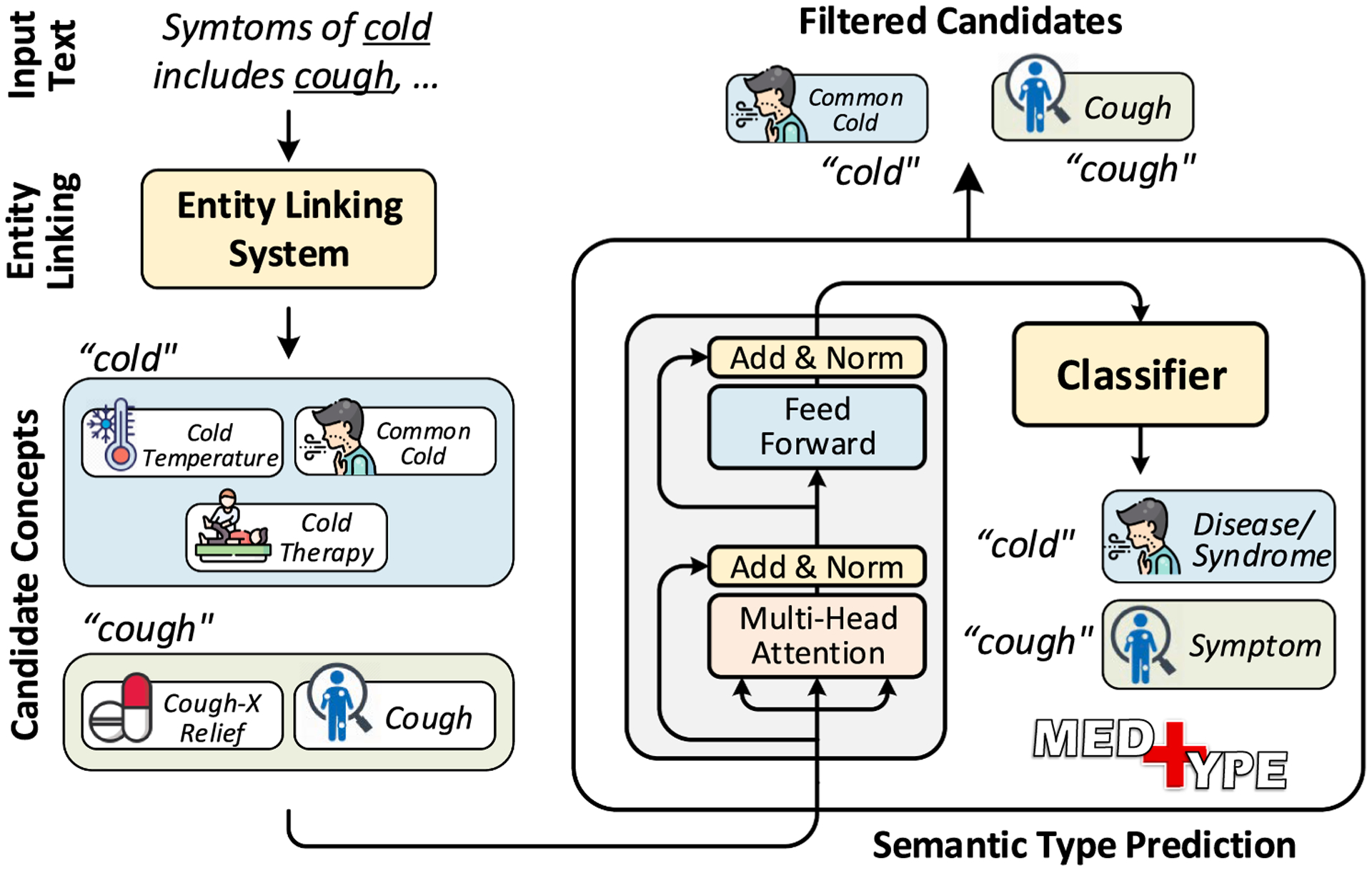
Overview of MedType. For a given input text, MedType takes in the set of identified mentions along with their list of candidate concepts as input. Then, for each mention, MedType predicts its semantic type based on its context in the text. The identified semantic type is used to filter out the irrelevant candidate concepts thus controlling overgeneration of candidates and improving medical entity linking. Please refer to Section 3 for details.

Broad-coverage information extraction from biomedical text faces dual challenges of (1) a breadth of dozens of information types and millions of candidate concepts that must be considered; and (2) resolving ambiguity even for known surface forms, long recognized as challenge for off-the-shelf information extraction tools [6] even while development of standalone disambiguation and linking models has progressed [37,38]. For instance, as shown in Fig. 2, *‘cold’* can refer to several distinct concepts such as *common cold*(disease), *cold temperature* (natural phenomena), or *cold brand of chlorpheniramine-phenylpropanol-amine* (pharmacologic substance). This ambiguity arising from *polysemy* and *homonymy* leads to overgeneration of candidate concepts, exacerbated by the breadth of potential information types of interest. Thus, including an additional step to prune irrelevant candidate concepts has the potential to improve entity linking performance by simplifying the final disambiguation step.

In this work, we formulate semantic type prediction and filtering as a standalone module MedType : $(C,m)\to C^{\prime }$, for integration into biomedical information extraction pipelines. The general type prediction and filtering process is as follows:

1. MedType takes in as input a medical entity mention *m* and a generated set of candidate concepts C={c_1,c_2,…,c_k}, each of which has one or more semantic types (here, drawn from the UMLS).
2. MedType consists of two steps: *MedType_Predict*: *m*→*t* ∈ *T*, where *T* is the set of all semantic types, and *MedType_Filter* : C→C'.
3. *MedType_Predict* takes the medical entity mention *m* and predicts the most likely semantic type *t* of the mention.
4. *MedType_Filter* takes the candidate set C and outputs a filtered set $C^{\prime }=\{c\_1^{\prime },c\_2^{\prime },\ldots c\_{k^{\prime }}^{'}\}$, such that *k*^′^⩽*k* and *c_*1^′^…*c_k*^′^ are all of the predicted semantic type *t*.

We further present a neural implementation of MedType as a standalone module which can be easily integrated into existing biomedical NLP pipelines. In Fig. 2, MedType predicts the given occurrence of *‘cold’* as referring to a disease, enabling pruning of the other candidates and resolving the ambiguity without the need of a dedicated disambiguation module. MedType utilizes recent advances in deep learning-based language modeling techniques [39,40] for encoding context to predict the semantic type of a mention. The overall semantic type filtering workflow and the architecture of MedType are shown in Fig. 2; details of the semantic type prediction task and MedType architecture are given in the following sections.

### 3.1. Information extraction problem definition

Formally, the task of information extraction is defined as follows. Let E={e_1,e_2,…,e_N} be a predefined set of entities in a knowledge graph and T=(w_1,w_2,…,w_|T|) be a given unstructured text with *n* tokens. The information extraction task involves identifying mentions {*m_*1*, m_*2, …*, m_k*} of the form *w_i*…*j* in T (mention detection phase) and mapping them to an entity e∈E (entity linking phase). Following prior work [41,42], we define E as the set of entities in the UMLS [10]. Most entity linking methods follow a two-step procedure: (1) Candidate Generation, which involves generating a probable set of candidates $C\_i=\left\{e^{i}\_1,e^{i}\_2,\ldots ,e^{i}\_l|e^{i}\_j\in E\right\}$ for each mention *m_i*, and (2) Disambiguation (often referred to as Word/Phrase Sense Disambiguation, or WSD), which involves choosing the highest-likelihood candidate concept $e^{i}\_j\in C\_i$.

### 3.2. Candidate pruning using semantic type

While many non-dictionary-based methods for medical entity linking have been proposed (e.g., [43,44]), the most frequently-used off-the-shelf tools [6,3] for broad-coverage biomedical information extraction (as well as many recent hybrid models [45–47]) rely heavily on dictionary lookup and sub-string matching. In the broad-coverage setting, the sheer number of medical concepts and prominence of lexical ambiguity among mentions due to *homonymy* and *polysemy* [19,20] leads to systematic over-generation of candidate concepts.

To alleviate this problem, we utilize an intermediate step of *semantic type filtering*, which takes in a generated candidate set C for a given mention *m* and outputs a filtered set $C^{\prime }\underline{\underline{\subset }}\;C$ based on the predicted semantic type of *m*. Fig. 2 illustrates this process: several irrelevant candidate concepts for the mention *cold* are pruned by identifying its semantic type of *Disease/Syndrome* in the given context. The semantic type of a mention is identified based on its usage in the text. For instance, in Fig. 2, based on its occurrence, the mention *cough* can be interpreted as a *symptom* rather than a *medicine*.

### 3.3. Mapping semantic types to groups

The semantic types in the UMLS Metathesaurus present two challenges for type prediction. First, each concept may have more than one semantic type (e.g., C0250873 *OX7-SAP* is both a *Pharmacologic Substance* and an *Immunologic Factor*). Second, type frequencies are strongly right-tailed: for example, 907,398 concepts are of type *Eukaryote*, while only two UMLS concepts have type *Carbohydrate sequence*; these differences are exacerbated by the sparsity of fine-grained types in entity linking datasets. To ameliorate both of these issues, we map the 127 semantic types in the UMLS Metathesaurus to 24 groups, as shown in Table 1. These groupings are derived from the UMLS semantic groups defined by [48], with additional use of *is-a* relationships to split too broad groups. We use these broader groups as the labels for multi-label semantic type prediction and filtering.

### 3.4. MedType architecture

MedType is a neural model for semantic type prediction in biomedical text, which is fully modular and can be included in any biomedical NLP pipeline. MedType takes in the input data of the form D=[(x_0,y_0),…,(x_N,y_N)] where *x_i* denotes the mention *m_i* and its surrounding context. The context comprises of the neighboring tokens in a window of size *k*, i.e., *Con*(*m_i, k*) = (*m_i*^−*k*^, …*, m_i*^−1^*, m_i*^−1^, …*, m_i*^*k*^) and *y_i* is the semantic type. Motivated by the ability to handle polysemous tokens and superior modeling capabilities of long range dependencies of Transformer-based models [49], we utilize a pre-trained BERT [40] encoder and fine-tune it for our type prediction task. In our experiments, we use BioBERT [50], an adapted BERT model for biomedical corpora. We give the mention with its context, i.e., (*m_i*^−*k*^, …*, m_i*^−1^, [men] *, m,* [*/*men] *, m_i*^−1^, …*, m_i*^*k*^) as input to the encoder. Here, the special tokens [men] and [/men] are meant for providing the positional information of the mention to the model. Finally, the embedding corresponding to [men] token is passed to a feed-forward classifier for the prediction of semantic types.

## 4. Novel datasets for medical entity linking

The availability of large scale public datasets helps to drive informatics research forwards [51–53]. However, curating large-scale biomedical datasets presents significant obstacles, including the expense and scarcity of relevant expertise, which largely precludes crowd-sourcing [23]; this is compounded in the case of medical records by the challenges of maintaining patient confidentiality and privacy [24]. To further medical entity linking research in light of these challenges, we present WikiMed and PubMedDS, two large-scale, automatically-created datasets for medical entity linking. We describe both the datasets in detail in the following sections.

### 4.1. WikiMed: Wikipedia-based medical entity linking corpus

#### WikiMed Construction:

**Fig. 3.**
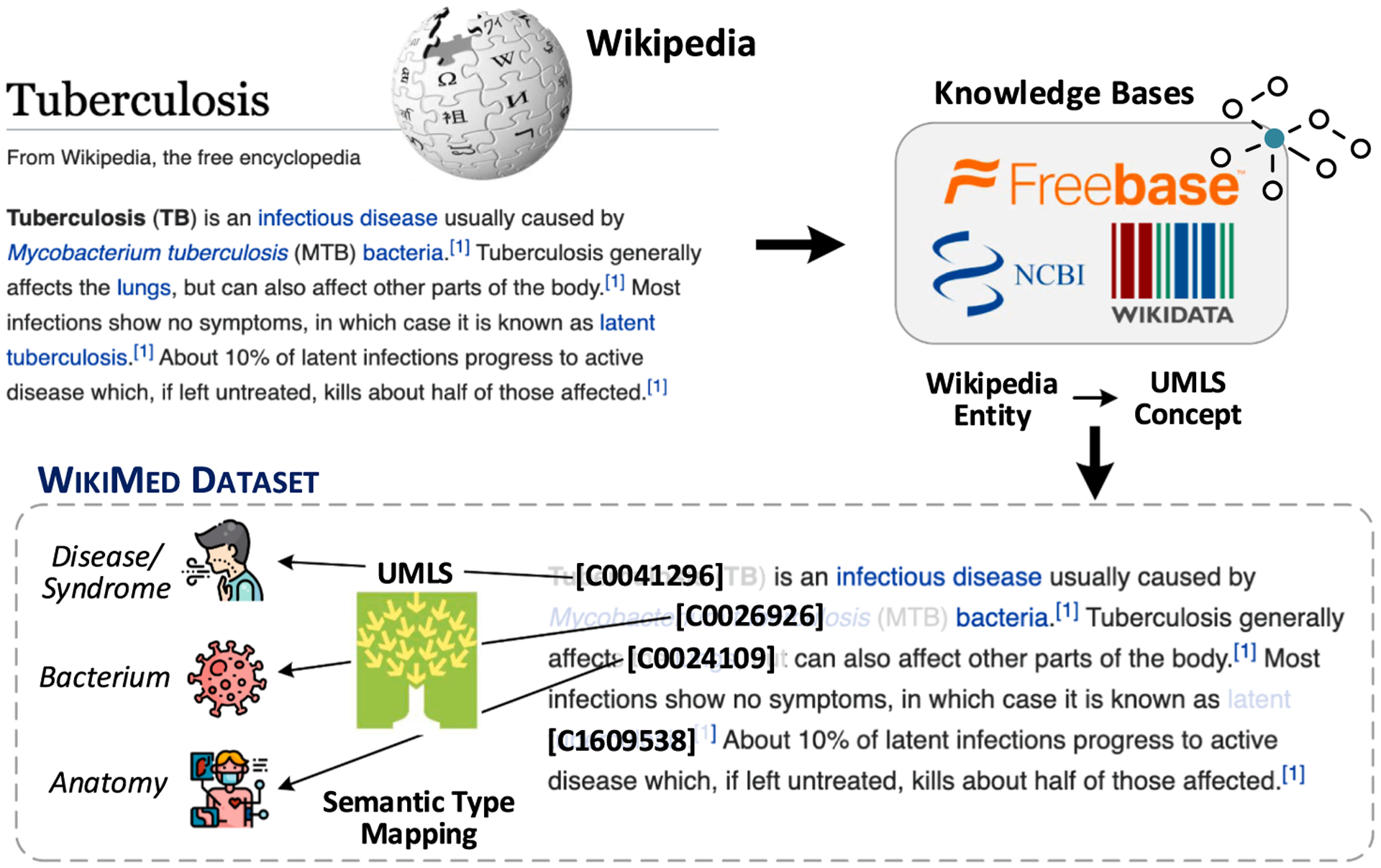
Constructing WikiMed from Wikipedia data. We map each linked mention in Wikipedia articles to a UMLS concept using mappings obtained from Freebase, Wikidata and NCBI knowledge bases.

The overall steps for creating WikiMed dataset are depicted in Fig. 3. Wikipedia, though not restricted to medical information, includes a large number of mentions of medical concepts that can inform entity typing models. We leverage that for constructing WikiMed dataset. Firstly, we extract the mapping of Wikipedia pages to UMLS concepts from several existing knowledge bases such as Wikidata [54], Freebase [55], and the NCBI Taxonomy [56]. This gives us a one-to-one mapping of approximately 60,500 Wikipedia pages to UMLS concepts. Since UMLS concepts are primarily biomedical in nature, this helps us identify the relevant Wikipedia pages for medical entity linking. Then, for each Wikipedia article, we linked those mentions to UMLS concepts. The Semantic Network (of UMLS) provides semantic types for each UMLS concept which we utilize for further reassigning mentions to semantic types. This results in a high-quality dataset for medical entity typing. Overall, our pipeline extracts around 1 million mentions spanning across 400 k Wikipedia articles. More details of the dataset are presented in Table 4. Although WikiMed contains web text on a variety of topics, we find that it helps to improve performance on entity linking in other domains as well as shown in Section 6.1.

#### WikiMed Quality:

The link structure of Wikipedia, which we utilized for creating the WikiMed dataset, is normally treated as ground truth in information extraction and natural language processing research [57–61]. While errors have been found in Wikipedia link structure [62,63], the average error rate of relational statements (including incorrect assertions and incorrect links) has been estimated to be around 2.8% [64], supporting the use of Wikipedia links as a sufficiently high-quality resource to yield accurate mappings. To assess the correctness of our medically-focused dataset, we randomly sampled 100 links from WikiMed for manual verification. Three authors (SV, DNG, RJ) reviewed each sample to assess (1) whether the annotated CUI (identified via automated mapping to the UMLS) was appropriate and (2) in cases of an incorrect CUI, whether the annotated semantic type was appropriate. After resolution of disagreements, we found a CUI-level accuracy of 91%, and a type-level accuracy of 95% in the 100 reviewed samples. As Wikipedia links are provided a priori in the page hypertext, and not all relevant mentions of an entity are marked with links, we did not assess either precision or recall of mention detection. Thus, while WikiMed is not appropriate for training or evaluating mention detection models, we find that it provides a high-quality silver standard resource for medical entity linking.

WikiMed is significantly larger than previous medical entity linking datasets: 3× larger than MedMentions [65], and 10× larger than the NCBI Disease Corpus [66]. Moreover, WikiMed also provides better coverage of entities from different semantic types than existing datasets, as shown in Table 2.

### 4.2. PubMedDS: Distantly-supervised biomedical entity linking corpus

#### PubMedDS Construction:

**Fig. 4.**
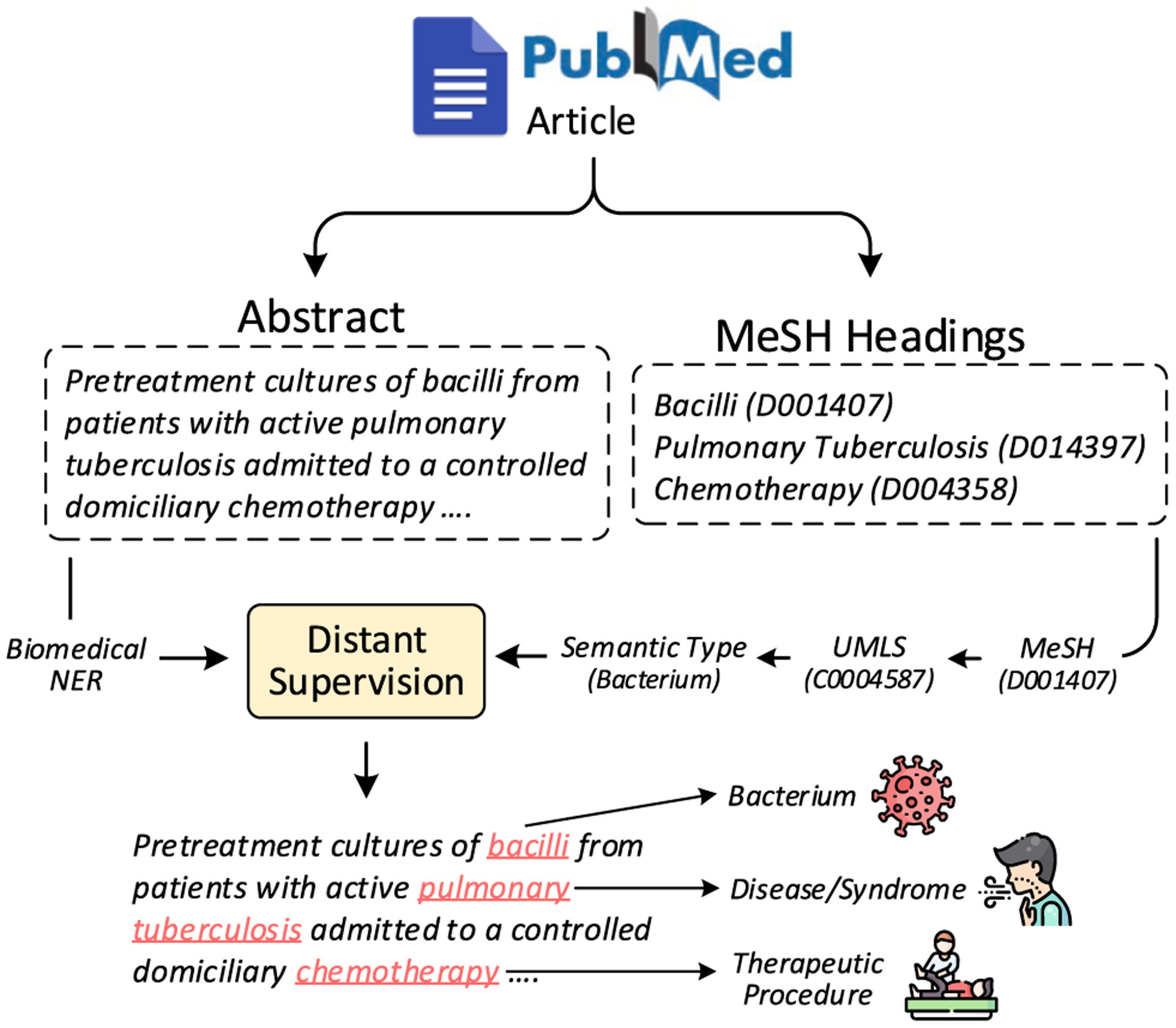
Constructing PubMedDS using distant-supervision on PubMed corpus. For each article, we apply biomedical NER on its abstract for obtaining relevant entity mentions which are then linked using supervision from MeSH headings of the article. Refer to Section 4.2 for details.

Distant supervision [67] enables automatic generation of training data and has been exploited for several tasks [68,69], including identifying potential mentions of medical concepts [70]. To create a large-scale training dataset for medical entity linking drawn from biomedical language, we use distant supervision on PubMed abstracts to generate PubMedDS. An overview of the entire process is summarized in Fig. 4. We first run a state-of-the-art biomedical NER model [42] on 20 million PubMed abstracts to extract its medical entity mentions. We then use the Medical Subject Headings (MeSH) tags assigned to each PubMed article to weakly link the extracted entity mentions to a MeSH concept. A mention is linked only when it exactly matches with the name of one of the provided MeSH headers. The UMLS provides mapping of MeSH headers to UMLS concept identifiers, which we utilize to get the semantic type of each linked mention from Semantic Network as done for mentions in WikiMed. Using this procedure, we created PubMedDS, a dataset with 58 M annotated mentions, which we utilize for pre-training MedType. The size of PubMedDS is around 164 times larger than the current largest medical entity linking dataset, MedMentions [65]. Next, we demonstrate that although PubMedDS is distantly-supervised, it has sufficiently high precision to serve as a valuable resource for medical entity linking research.

#### PubMedDS Quality Analysis:

Distant supervision enables large-scale text annotation but can produce noisy data [71]. In order to assess the quality of PubMedDS as a dataset for medical entity linking, we identified the subset of documents overlapping with three manually-annotated datasets using PubMed abstracts: MedMentions [65], NCBI [66], and Bio CDR [72]. All PubMed documents annotated in these three datasets were included in PubMedDS. This allowed us to compare the precision and recall of our distantly-supervised mentions to manual annotations. The results of this analysis are reported in Table 3. Reflecting on the strict requirements for linking a mention in our dataset (identification with a NER tool and exact match to a provided MeSH header), we find that PubMedDS omits many of the true mentions in these documents, but the vast majority of included mentions are annotated correctly (precision of around 84%). Thus, while PubMedDS would not be appropriate for training medical mention detection (NER) models, its annotations are of high quality for training entity type prediction and disambiguation models.

## 5. Experimental evaluation

Our work makes three distinct contributions to broad-coverage information extraction research: (1) a modular formulation of the semantic type prediction task, which can be easily integrated into any pipelined approach; (2) our MedType model for semantic type prediction; and (3) our novel datasets for biomedical entity linking research. We thus performed two types of experimental evaluations leveraging four benchmark datasets for biomedical information extraction (detailed in Section 5.1).

### Semantic type prediction:

We first evaluated MedType as a stand-alone model for semantic type prediction, comparing it against recent type prediction models (detailed in Section 5.2) to measure the specific improvements yielded by our approach. We used the gold mentions annotated in each dataset directly, without use of a mention detection model. The label for each mention was identified by mapping its annotated CUI to its semantic type(s) in the UMLS, and from there to one or more of our 24 semantic groups (described in Section 3.3). We trained each type prediction model to predict these classes, using the training portion of each dataset and evaluating on the test set.

In addition, we measured the impact of our novel entity linking datasets: WikiMed and PubMedDS by pretraining our best performing model, MedType on each dataset individually and on both together prior to training on each of the four evaluation datasets, and comparing type prediction performance to using MedType without pretraining.

### Information extraction:

We then evaluated the impact of using semantic type filtering as part of five widely-used biomedical information extraction pipelines (detailed in Section 5.3). To evaluate the semantic type filtering module and our MedType implementation separately, we experimented with three approaches for semantic type prediction:

- **Oracle (fine):** To evaluate the maximum possible improvement from type-based pruning of candidate concepts, we experimented with an oracle model which always filters the candidate set of entities to entities of the same type as the gold standard CUI. The *Fine* oracle filters based on the 127 original types in the UMLS, to control for effects of semantic grouping.
- **Oracle (coarse):** Our *Coarse* oracle uses the 24 semantic groups defined in Section 3.3, to represent an upper bound of what can be achieved using our type prediction models.
- MedType: Finally, for a practical evaluation aligned with real-world use, we incorporate both MedType and its strongest competitor type prediction model into the information extraction pipelines to perform semantic type filtering.

Under each of these settings, we integrate semantic type prediction into the information extraction pipeline as follows:

1. Run biomedical information extraction tools to identify (1) mentions of medical concepts in a document; and (2) a ranked list of candidate CUIs for each mention.
2. Use one of the above semantic type prediction approaches to predict the type of each mention, and filter the list of candidate CUIs to only CUIs of that type.
3. Return the highest-ranked CUI in the filtered candidates as the final entity linking prediction.

### 5.1. Datasets

In our experiments, we evaluate the models on four benchmark datasets: the NCBI Disease Corpus [66], Bio CDR [72], ShARe [73], and MedMentions [65] for medical entity linking. These datasets span across different text genres, such as biomedical research articles and Electronic Health Records (EHR), and information domains, allowing us to evaluate the generality of MedType across diverse domains. The dataset statistics and the semantic type distribution are presented in Table 4 and Table 2 respectively. Below, we provide a short description of each dataset.

- **NCBI:** The NCBI Disease Corpus [66], which we refer to as NCBI for brevity, consists of 793 PubMed abstracts annotated with disease mentions and their corresponding concepts in the MEDIC vocabulary [74].
- **Bio CDR:** The CDR corpus [72] consists of 1,500 PubMed abstracts annotated with mentions of chemicals, diseases, and relations between them. These mentions were normalized to their unique concept identifiers, using MeSH as the controlled vocabulary.
- **ShARe:** The ShARe corpus [75] is a collection of de-identified clinical notes, which was used for a series of NLP shared tasks. We use the subset used in a 2014 shared task [76], consisting of 431 documents annotated for disorder mentions and grounded to SNOMED CT.
- **MedMentions:** The MedMentions data of [65] consists of 4,392 PubMed abstracts annotated with several biomedical mentions. Each mention is labeled with a unique concept identifier and a semantic type using the UMLS as the target ontology.

### 5.2. Type prediction baselines

We compare MedType against four recent neural entity typing methods. **AttentionNER** [77] utilizes attention mechanism for extracting relevant information from the context of a mention for type prediction. **DeepType-FC** and **DeepType-RNN** are two neural network based models proposed by [22] for entity typing. **Type-CNN** [78] is another neural approach which utilizes CNNs for modeling the global context of a mention for type prediction. **MedNER** [36] uses NLM and dictionary mapping to predict semantic type of medical mentions.

### 5.3. Biomedical information extraction tools

We integrate MedType into five widely-used tools for biomedical information extraction, each of which performs mention detection (NER) and produces a ranked list of candidate CUIs for each mention. Below, we describe each of them in brief.

- **MetaMap** [6] leverages a knowledge-intensive approach based on symbolic NLP and linguistic techniques to map biomedical mentions in text to UMLS concepts. MetaMap was developed for indexing scientific literature.
- **cTAKES** [3] uses a terminology-agnostic dictionary look-up algorithm for mapping named entities to UMLS concepts. We utilize the Clinical Pipeline of cTAKES augmented with LVG Annotator^2^. cTAKES was developed for analyzing clinical text.
- **MetaMapLite** [79] re-implements the basic functionalities of MetaMap with an additional emphasis on real-time processing and competitive performance.
- **QuickUMLS** [41] is a fast, unsupervised algorithm that leverages approximate, dictionary-matching techniques for mapping biomedical entities in text. QuickUMLS was developed as a general-purpose tool and evaluated on consumer-generated texts [41].
- **ScispaCy** [42] builds upon the robust spaCy library [80] for several biomedical and scientific text-processing applications such as parsing, named entity recognition, and entity linking. ScispaCy was developed primarily for analyzing scientific literature.

We do not use the recent CLAMP [16] system in our experiments, as it does not provide access to a generated list of candidates for a mention prior to the disambiguation step.

### 5.4. Evaluation metrics

For semantic type prediction, which we model as a multi-label classification problem, following [81,82], we use the area under the Precision-Recall curve (AUC) as our evaluation metric.

For entity linking, we evaluate the performance using F1-score for two metrics. In (1) *Exact*_*mention*_*id*_*match* (**Exact**), true positives are only those samples where both the predicted mention bounds and entity concept identifier exactly match the annotation. This is directly adopted from TAC KBP 2013^3^. In (2) *Partial*_*mention*_*id*_*match* (**Partial**), a weighted score is assigned to predicted mentions based on the amount of overlap with annotated mention bounds and entity id match. Following [83], for mention matching, the number of overlapped characters between system generated mention and a ground-truth mention is considered. All the scores are computed using an open-source entity linking evaluation toolkit^4^.

### 5.5. Implementation details

#### Online Demo & medtype-as-service:

Along with providing a step-by-step guide for reproducing all the results reported in the paper, we also provide code for running an online demo of MedType. We also provide a scalable implementation of MedType called medtype-as-service which is based on *bert-as-service* [84] for processing thousands of documents simultaneously.

#### Hyperparameters:

We use pre-trained weights of BioBERT [50] for initializing BERT component of MedType. MedType is implemented using HuggingFace Transformers library [85]. For training, we utilize Adam optimizer [86] with a learning rate in range (10^−3^, 10^−5^). The window size of context (*k*) is chosen from {48, 64, 128}. The best hyperparameters were selected based on the performance on the validation split of the datasets. We use the default hyperparameters for all the entity linkers and components of MedType. A grid search over the validation split was performed for deciding a threshold for each semantic type from the range of (0.001, 1). The area under the Precision-Recall curve (AUC) was used for choosing the best threshold.

#### Training Details:

All training was performed on NVIDIA-GTX 1080Ti GPUs. Each training epoch of MedType takes from 5 min to 2 days depending on the size of the dataset. The models are trained for multiple epochs until the validation performance starts to degrade. In terms of number of parameters, MedType has around 110 million parameters (same as BERT-base model).

## 6. Results

Medical information extraction is a complex process, with multiple points of evaluation and multiple types of impact from any new contribution. We present results for four specific questions that examine the impact of semantic type filtering with MedType:

Q1. How effective is MedType for semantic type prediction, and what is the impact of our novel datasets? (Section 6.1)
Q2. Does incorporating MedType in existing entity linking systems help the overall pipeline? (Section 6.2)
Q3. What specific successes do we see from combining MedType, WikiMed, and PubMedDS, and what are remaining challenges? (Section 6.4)
Q4. How much does semantic type-based filtering help prune irrelevant candidates? (Section 6.5)

### 6.1. MedType is State-of-the-art for medical semantic type prediction

The first step in our evaluation is a modular investigation of the semantic type prediction task on its own. In this section, we compare MedType against the baseline methods detailed in Section 5.2 for semantic type prediction. We also evaluate the effectiveness of utilizing WikiMed and PubMedDS datasets for the task. For quantifying the benefit of our proposed method and datasets, we report the performance of MedType trained under different settings, as defined below.

- MedType **(MT)** denotes MedType trained on the training split of the corresponding datasets.
- **MT** ← WikiMed refers to the model first trained on WikiMed and then fine-tuned using the training data.
- **MT** ← PubMedDS similar to T ← WikiMed, indicates MedType first trained on PubMedDS and then fine-tuned on the training data.
- **MT** ← **Both** denotes the combined model which utilizes both the proposed datasets. It concatenates BERT encoding from T ← WikiMed and T ← PubMedDS models and passes it to a classifier which is trained using the training dataset.

Semantic type prediction results are presented in Table 5. We find that MedType outperforms all the baselines on three of the four evaluation datasets when trained only on the training split. Compared to the best performing baseline, we obtain a gain of 0.2, 0.7, and 9.1 AUC on Bio CDR, ShARe, and MedMentions respectively. MedMentions contains a much greater diversity of semantic types than other datasets (as shown in Table 2). Thus, obtaining a large improvement on it indicates that MedType is more suited for handling large set of types compared to the baseline methods.

Further, we find that utilizing our novel datasets WikiMed and PubMedDS yields considerable gain in performance. On average, we obtain an increase in AUC of 1.7 from WikiMed alone, 3.9 from PubMedDS alone, and 4.5 from using both, across all datasets. The combined model which allows to incorporate the benefits from both the corpora gives the best performance. This shows that both the datasets contain complementary high-value information for semantic type prediction.

### 6.2. MedType Consistently improves overall information extraction performance

The primary goal of our study is to investigate the impact of adding a semantic type prediction module to the medical information extraction pipeline. In this section, we evaluate the impact of MedType on biomedical information extraction when integrated with the tools detailed in Section 5.3. Table 6 reports the results for the *Exact*_*mention*_*id*_*match* and *Partial*_*mention*_*id*_*match* metrics, as described in Section 5.4.

As discussed in Section 5.1, the NCBI, Bio CDR, and ShARe datasets were annotated for specific categories of medical concept mentions (e.g., diseases and disorders only); concept mentions outside of these categories were excluded from annotation. By contrast, the information extraction tools we experimented with were all preconfigured for broad-coverage extraction of all types of medical information. Thus, the set of predicted medical concept mentions output by any one of our toolkits could include concepts of a type excluded from dataset annotation—predictions which we are therefore unable to evaluate. To avoid including these mentions in our evaluation, we filtered the output of each toolkit for a given dataset to the semantic types included in that dataset’s annotation (e.g., disease mentions only for the NCBI Disease Corpus). We determined the semantic type of predicted concept mentions using the final CUI produced as the top-ranked candidate after processing with the full information extraction pipeline (including semantic type prediction, when used). Thus, if the top-ranked candidate for a given mention was of an excluded type when using an unmodified entity linker, that mention would be excluded from evaluation (informing both mention detection and entity linking evaluation); however, if the introduction of semantic type filtering removed that top-ranked candidate in favor of a lower-ranked candidate of a type *included* in dataset annotation, the mention would be included in evaluation.

We compare MedType against the two oracle approaches described in Section 5, as well as against the best-performing baseline from Section 6.1. For each information extraction system, we report its default performance along with the change in scores when adding different type-based candidate filtering methods. The results for MedType are obtained after pre-training on WikiMed and PubMedDS datasets, based on our findings in Section 6.1.

Across most information extraction tools and datasets, MedType yields a substantial improvement in performance, and it consistently matches or outperforms Type-CNN, the best prior method for type prediction. Notably, in no situation does MedType degrade performance; thus, the results indicate that including a type-based filtering step enhances information extraction systems in most cases. (See Section 7.5 for a discussion of the differences between performance of individual information extraction tools.) The gain with MedType is comparable to improvement with using an oracle, indicating that MedType is reliable enough to use off-the-shelf. The results also show that there is not much difference in performance of Oracle (Fine) and Oracle (Coarse). This justifies our choice of working with 24 semantic groups rather than the 127 semantic types defined in the UMLS Metathesaurus.

We used paired bootstrap significance testing [87] for validating statistical significance (*p* < 0.01) of improvements from MedType compared to the default pipeline and the top performing baseline performance. Our results clearly support the central thesis of this work, that pruning irrelevant candidate concepts based on semantic type helps improve medical entity linking.

### 6.3. MedType improves entity linking performance

The evaluations described in Section 6.2 account for both mention detection—which semantic type filtering can affect by removing all candidates for a mention, leading to its exclusion—and entity linking. We therefore isolated the effect of MedType on the entity linking portion of the information extraction pipeline alone by restricting our analysis to only predicted concept mentions overlapping with gold annotated mentions, and calculating the *Partial*_*mention*_*id*_*match* F-1 metric (detailed in Section 5.4) on this subset. Table 7 reports results for ScispaCy (the best-performing information extraction tool) on all four evaluation datasets.

Baseline performance with ScispaCy is 7–10 points higher in this more restricted evaluation, as compared to Table 6, reflecting the additional challenges of mention detection which go into the overall evaluation. Semantic type filtering leads to similar improvements for NCBI and Bio CDR in this setting, but noticeably larger improvements on ShARe and MedMentions, demonstrating that overall information extraction improvements from semantic type filtering are coming primarily from the entity linking portion of the pipeline.

### 6.4. Gains and challenges of MedType, WikiMed, and PubMedDS

#### PubMedDS and WikiMed yield large improvements for rare types:

As observed in Section 6.1, pretraining MedType on WikiMed and PubMedDS led to substantial increases in semantic type prediction performance. In this section, we investigate which types of medical concept mentions were improved the most from this pretraining step. For this, we report the F1 score of MedType, MT ← WikiMed, MT ← PubMedDS and MT ← Both models (as defined in Section 6.1) across all semantic types on all the datasets. The overall results are summarized in Table 8. In general, we find that performance improves across all semantic types as we utilize additional corpora, but the maximum gain is obtained on types which have less coverage in the training split. For instance, on types such as *Pathological Function* and *Sign or Symptom* in the NCBI Disease Corpus, the F1 score jumps from 0 to 80 and 83.3 respectively. Thus, the broad coverage of medical concept types in WikiMed and PubMedDS, combined with their large scale, helps to fill in the gaps of semantic types that are not well-represented in the evaluation datasets directly.

#### Error analysis of MedType:

To gain insight into further opportunities for improvement in semantic type prediction, we analyzed MedType errors in the validation split of the MedMentions dataset when using our best performing model, which is pre-trained on both WikiMed and PubMedDS datasets. As reflected by Table 5, MedType is able to identify the correct semantic type in the majority of cases. However, as Table 8 shows, performance is not uniform across semantic types; e.g., *Devices*, *Finding*, *Occupations*, and *Phenomena* (all involving fairly common words) remain particularly challenging in these data. Table 9 shows the semantic types most commonly confused with one another, in many cases, we see mispredictions of more abstract types such as *Objects*, *Concepts* & *Ideas*, and *Functional Concepts*, regardless of gold semantic type. Thus, there is still significant scope for improvement on this problem.

### 6.5. Impact of semantic type prediction on candidate generation

The preceding sections have shown that semantic type filtering consistently improves entity linking performance when using the candidate scoring methods provided in each of our evaluated information extraction tools. However, candidate ranking and disambiguation are active areas of research [18,37], and the modular nature of both our MedType model and the semantic type filtering task makes it easy to incorporate type filtering into any entity extraction pipeline. We therefore investigated the impact of semantic type prediction in filtering out over-generated candidate concepts, in order to understand how type filtering simplifies the final disambiguation task.

#### Semantic type-based pruning consistently reduces the candidate set size.

**Fig. 5.**
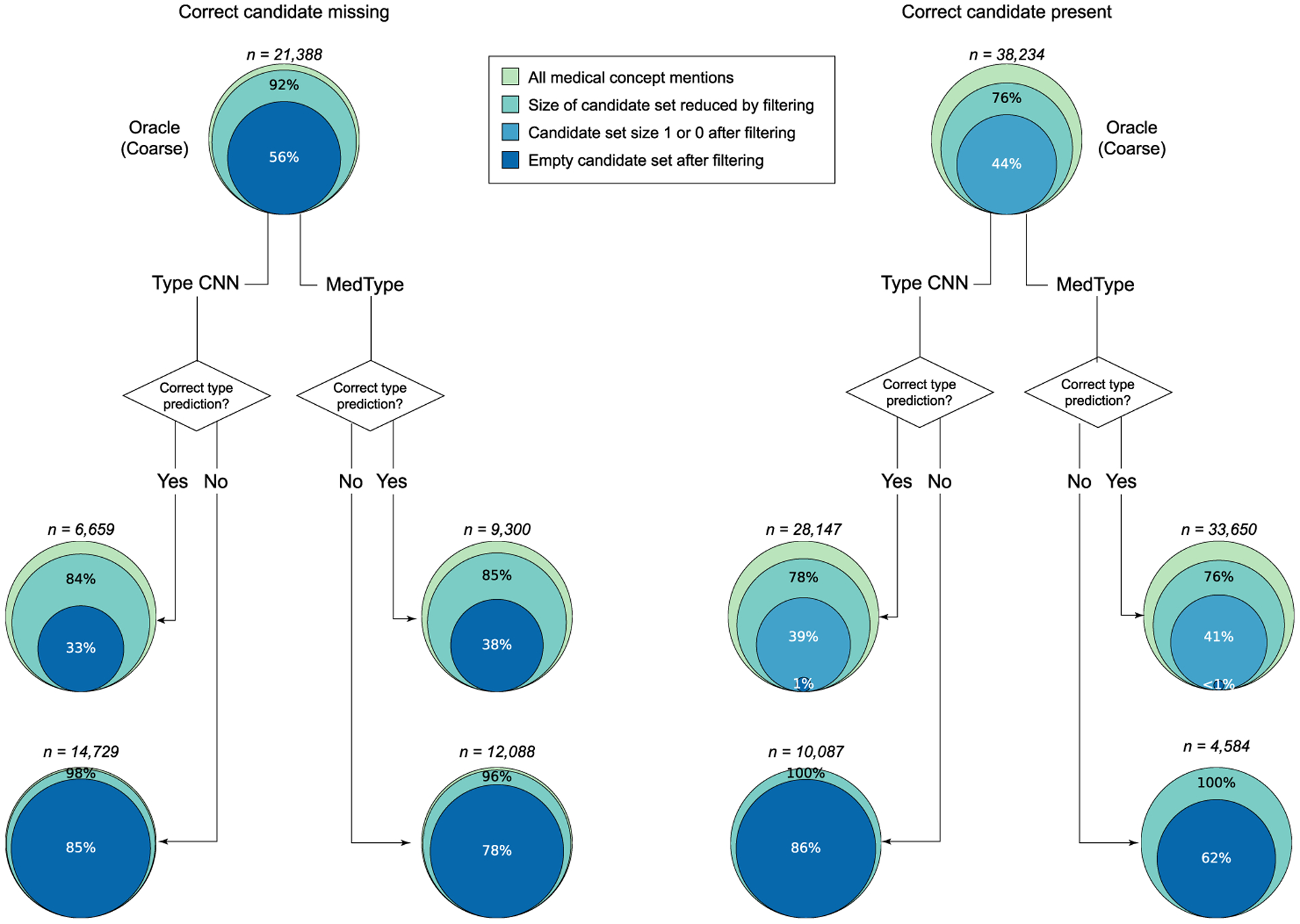
Outcomes of semantic type filtering in MedMentions data, in terms of reduction in candidate set size. All results are reported using the best-performing information extraction model (ScispaCy). Top graphs display candidate set reduction using oracle type filtering, broken down into whether the correct candidate was included in the list generated by ScispaCy. Bottom graphs illustrate corresponding outcomes from MedType and the strongest type prediction baseline (Type CNN), broken down by whether the predicted type was correct. The number of samples each graph displays is provided, along with the percentage of these samples included in each reduction category.

Fig. 5 illustrates the outcomes of type-based pruning on the candidate set sizes for both the 38,234 samples in the MedMentions test set where ScispaCy included the correct CUI in its candidate set and the 21,388 where it did not. Oracle type information, representing the upper bound of what type-based pruning can achieve, reduces the candidate set size in over 75% of “Correct candidate present” cases at the coarse level, and directly solves the sense disambiguation problem in 44% of cases. Fine-grained typing, not shown in Fig. 5, only slightly improves these results—candidate set size reduction in 81% of cases, full disambiguation in 54%—while significantly complicating the type prediction problem, further supporting our choice of coarse labels for MedType. MedType, in turn, achieves most of the reductions in candidate set size yielded by oracle information, and the performance improvements shown in Table 6 clearly demonstrate the practical gains from this filtering. MedType further considerably reduces the number of type mispredictions over the best baseline, as seen also in Table 5.

#### MedType can help improve the full extraction pipeline.

**Fig. 6.**
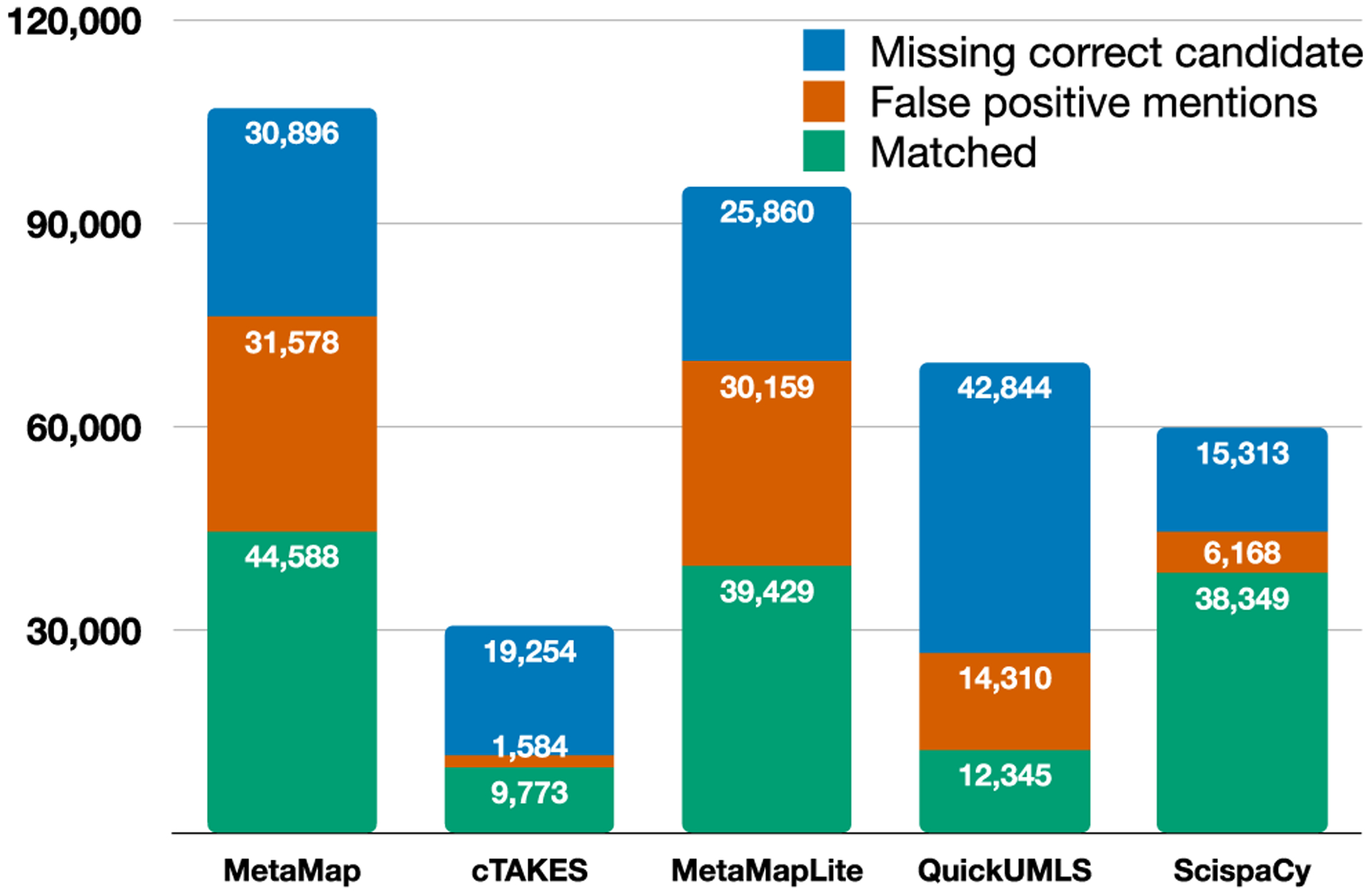
Error analysis of output predictions from all information extraction tools on the MedMentions test set (annotated set size: 70,405 mentions). False positive mentions are spurious entity spans extracted by the tools; Missing correct candidate cases indicate exclusion of the correct entity from the returned candidate list. Matched indicates that neither of these errors were present. Refer to Section 6.5 for details.

Failures can occur at all three stages of entity extraction: mention detection (NER), candidate generation, and disambiguation. Fig. 6 illustrates the number of medical concepts extracted by the information extraction tools we used in the MedMentions test set, broken down into (1) false positive mentions, where the mention detection stage of the pipeline produced a false positive entity span; (2) missing correct candidates, where the candidate generation phase of the pipeline did not include the correct entity in the candidate list; and (3) matches, where the tool found a valid span and included the correct entity in the candidate set. The five tools evaluated varied widely in the number of entities output, but in all cases include a significant number of both mention detection and candidate generation errors. In addition to MedType’s utility in reducing candidate set sizes, which allows for broader-coverage candidate generation methods, we also observe that in all cases where a false positive mention was produced, MedType classified it as a *None* type; this indicates clear utility in incorporating MedType as a component of any system to filter out false positives in NER.

#### Degree of candidate set size reduction from semantic type filtering.

**Fig. 7.**
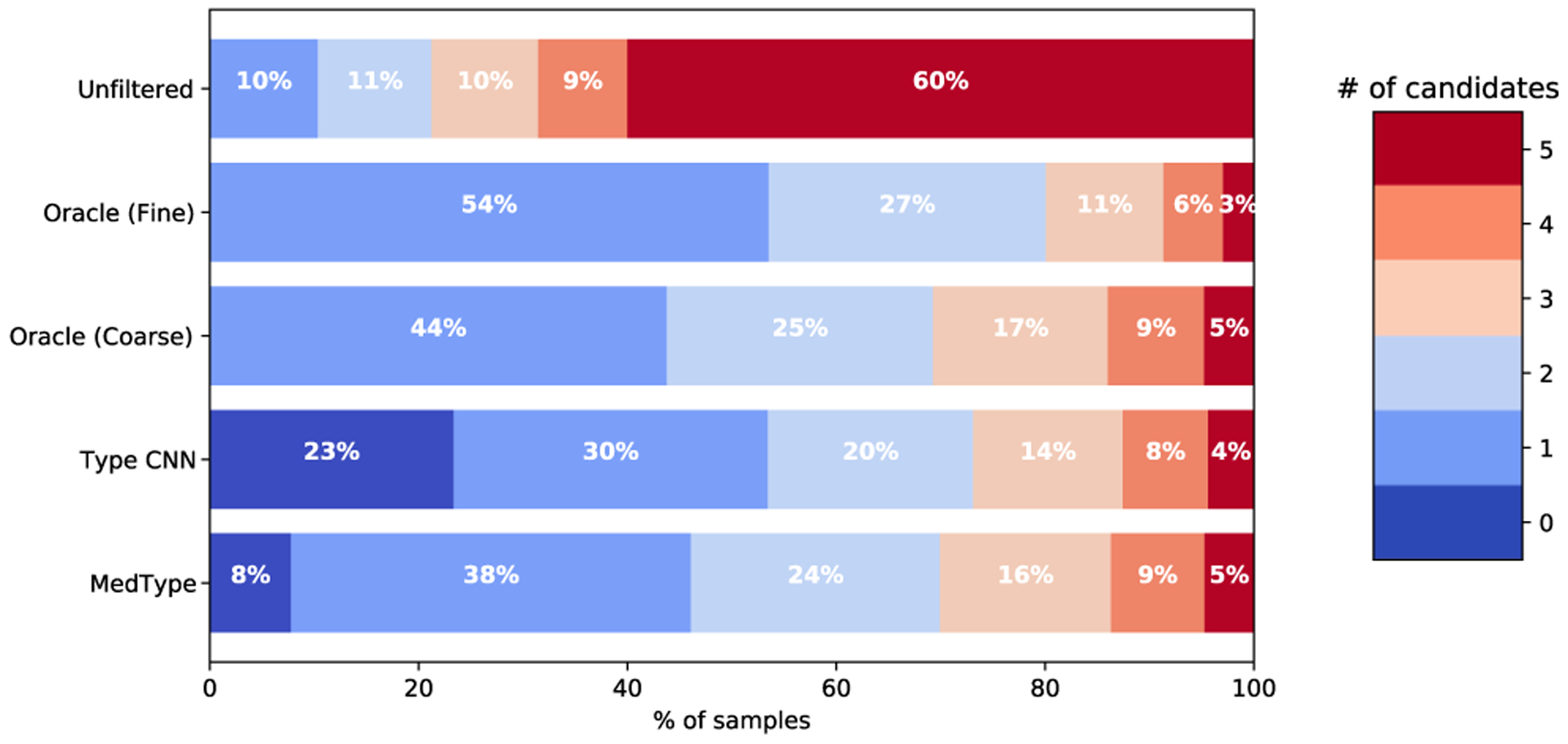
Distribution of candidate set sizes in MedMentions using ScispaCy, comparing unfiltered concepts to candidate sets filtered using semantic type prediction strategies. Only mentions predicted by ScispaCy that included the correct CUI in the candidate set are included. Larger bars to the left-hand side of the figure indicate greater reductions in candidate set size.

Fig. 7 expands the analyses presented in Fig. 5 to show the detailed distribution of the candidate set sizes within the predicted samples of MedMentions that included the correct candidate, comparing oracle type filtering strategies to MedType and the best type prediction baseline. ScispaCy, presented here as the best-performing information extraction tool on MedMentions, limits its output candidate set to 5 by default; however, all tools used displayed similar behavior in our experiments.

## 7. Discussion

We have demonstrated that semantic type filtering is a valuable addition to NLP pipelines for broad-coverage biomedical information extraction. We discuss broader impacts of MedType in biomedical NLP in Section 7.1, and other approaches to semantic type filtering in Section 7.2. We further highlight the contributions of our novel WikiMed and PubMedDS datasets for biomedical concept normalization research in Section 7.3, and note potential effects of biased data in Section 7.4. Finally, we discuss two further implications of our findings for continued research on this important use case: the choice of information extraction tool for a given setting (Section 7.5), and opportunities for further research synthesizing semantic type prediction and disambiguation (Section 7.6).

### 7.1. Broader applicability of MedType in biomedical NLP

Identifying mentions of biomedical concepts in text is one of the fundamental building blocks of biomedical NLP. As a result, a wide variety of highly heterogeneous methods have been developed to perform concept identification [88]. As a fully modular component which takes as input a set of candidates and returns a set of candidate as output, MedType can be easily incorporated into any type of medical concept recognition system that uses a set of candidate concepts. Such systems are key elements of NLP pipelines for diverse applications, such as adverse drug event detection [89], biosurveillance [90], and patient phenotyping [91]. Morever, many biomedical NLP applications that do not use concept-level mapping nevertheless make use of coarse-grained type information [92,93], which the modular type prediction component of MedType is well positioned to enhance. MedType’s role in refining and organizing medical information in text thus makes it a valuable addition to a wide variety of biomedical NLP pipelines, and its fine-tuning process can be easily used to adapt it to any dataset.

### 7.2. Generalizability and other approaches to semantic type filtering

Beyond alignment to the UMLS and other controlled vocabularies, biomedical NLP systems often employ custom typologies for specific applications, such as in analyzing radiology notes [94] or functional status information [95]. As seen in our experiments without pretraining, MedType can be trained to predict the semantic types of a dataset using a relatively small amount of data (i.e., hundreds of documents). Thus, MedType could be deployed as an element of NLP pipelines with custom typologies as well, via an intermediate step of training the type prediction model on the task-specific dataset.

More broadly, semantic type filtering as presented here is not specific to our MedType implementation; a variety of approaches could be used within the general framework described in Section 3. Past work has leveraged rule-based and lexical approaches for semantic type prediction [6,94], or incorporated semantic type prediction as one element of a larger joint neural system [36]. MedType serves as a strong baseline for additional research in this area.

### 7.3. WikiMed and PubMedDS are valuable resources for biomedical concept normalization research

The expense and difficulty of producing large-scale datasets is a major limiting factor in biomedical NLP research. This is particularly the case for the labor-intensive task of annotating datasets for biomedical concept normalization, where information density is high and there are thousands of candidate concepts to choose from in the annotation process. The WikiMed and PubMedDS datasets introduced in this work are a step towards alleviating this problem, presenting millions of annotated concept mentions with a high diversity in semantic type coverage. While these datasets were automatically created and therefore subject to noise from the link mapping process (WikiMed) and from distant supervision (PubMedDS), our evaluation of them shows the annotations to be a high-quality silver standard, which can serve as a valuable resource for further research on semantic type prediction and biomedical concept normalization.^5^

### 7.4. Potential effects of biased data on MedType and novel datasets

The effects of biased data and algorithms in producing biased AI systems (including medical AI systems) is an important and rapidly-growing area of inquiry [96,97]. While MedType is not directly predicting sensitive information related to patients, or decisions about their treatment, it is nonetheless worth noting potential sources of bias that may be reflected in the outcomes of this study. Two interrelated types of bias are important to discuss: demographic bias (e.g., racial or gender bias) and statistical bias (in the sense of modeling the characteristics of one dataset over another). One major contributing factor to demographic bias in NLP systems is a lack of representatively diverse data; by learning the characteristics of data produced by a subset of the population, the resulting models are less effective in more diverse settings [98,99]. A significant portion of biomedical NLP research (including many of the datasets used in this article) relies on PubMed—which reflects racial disparities in scientific funding and publication [100]—and Wikipedia—which exhibits both racial and gender biases in the presentation of information [101,102]. These biases thus have the potential to be propagated in terms of the different sets of language in which NLP models will be most effective. From a more statistical sense, models trained on one genre of text (such as Wikipedia) generally show some performance degradation when applied to text from other genres (such as PubMed). Investigating potential biases in biomedical NLP systems for information extraction is an important direction to continue in future work.

### 7.5. Contribution of semantic type filtering by information extraction toolkit

While our results show consistent improvements in information extraction performance from integrating semantic type prediction, the effect size varies from toolkit to toolkit and genre to genre. For example, improvements in MetaMap performance are 1% or less for NCBI, Bio CDR, and ShARe, while QuickUMLS performance noticeably improves on all datasets but NCBI, and ScispaCy and MetaMapLite show large improvements from semantic type filtering across the board. These differences are in large part attributable to differences in the size of the candidate set produced by each toolkit; for example, cTAKES, which sees small relative improvements from type filtering, produces the fewest average candidates per mention of the tools we used, while ScispaCy (as illustrated in Fig. 7) produces its built-in maximum of 5 candidates for the majority of samples analyzed. This indicates that revisiting candidate generation strategies, using semantic type filtering to balance out more permissive candidate generation, is a worthwhile direction for improving coverage in biomedical information extraction.

### 7.6. Opportunities for disambiguation research using semantic type filtering

Disambiguating the candidate concepts produced by medical entity extraction pipelines has been a long-standing area of research, with several tools developed to integrate with existing pipelines. The YTEX suite of algorithms [103,104] extends both MetaMap and cTAKES with a disambiguation module that helps to reduce noise considerably, although [105] found that it often over-filtered correct concepts. There has also been significant research in recent years on developing standalone models for disambiguation, using co-occurrence and feature-based approaches [106–108] as well as neural models [37,109]. Medical concept normalization more broadly has also become an increasing research focus [38,15], with significant opportunities for disambiguation research [21].

MedType, and the semantic type filtering task more broadly, can be easily combined with any of these approaches to create a multi-stage filtering strategy for the disambiguation stage of the information extraction pipeline. MedType performs coarse filtering to a high-confidence set based on predicted type, a key step for narrowing down over-generated candidate sets in both open-ended deep learning systems and dictionary-based pipelines built for broad coverage; disambiguation methods can then perform a fine-grained selection of the correct candidate to further improve entity linking performance. We highlight this as an important direction for future work on medical entity linking.

### 7.7. Limitations of this study

MedType consistently improves the performance of the medical entity linking systems we evaluated. However, this study has some limitations that can help to guide further research on medical entity linking methods. While our use of coarse-grained semantic types simplified the type prediction task and removed the issue of multiple valid types for UMLS concepts, these semantic groups can be overly broad in practice (e.g., combining symptoms and diagnoses into a single category) and may be qualitatively undesirable. Our fine-grained oracle results in Table 6 also showed frequent improvement over the coarse-grained oracle, particularly in the heterogeneous MedMentions dataset, suggesting further potential improvement from a more granular type prediction system.

In addition, while MedType helps to correct for candidate generation errors by pruning out all candidate concepts of the wrong type, it cannot identify a candidate that was not generated in the first place. Similarly, a candidate selection algorithm that improperly scores candidate concepts within a single semantic type will not be affected by MedType. Future research can leverage the value of semantic type filtering to take advantage of broad-coverage candidate generation approaches to improve recall, and fine-grained candidate scoring algorithms focusing on specific semantic types to improve precision.

For application purposes in biomedical settings, explainability and system accountability are often of high importance. Providing explanations for the opaque outputs of deep neural network models in medical settings remains a significant challenge [110], and there is an active debate over how explainable such models can be [111]. Providing insight into MedType successes and failures, and options for users to adjust system parameters for their specific settings, will be an important part of supporting broader adoption of biomedical NLP technologies like MedType.

Finally, our results are necessarily limited by the homogeneity of some of our datasets. Of the evaluation sets, only MedMentions includes samples of all semantic types; our picture of MedType’s impact is thus incomplete for other PubMed data or for clinical language.

## 8. Conclusion

Broad-coverage information extraction from biomedical text is an important application area for biomedical NLP tools, and one which poses significant challenges in the scale and diversity of information to extract. To help address these challenges, we introduced semantic type prediction as a modular component of biomedical information extraction pipelines, and presented MedType, a state-of-the-art neural model for semantic type prediction. We demonstrated that semantic type prediction measurably improves information extraction performance on four benchmark datasets from different genres of text and types of information, and that these improvements are observed consistently when integrating type prediction into five commonly-used tools for biomedical information extraction. We further presented two new, automatically-created datasets, WikiMed and PubMedDS, which are significantly larger than any previous resources for medical entity linking research. While the automated annotation processes to create these datasets introduced some noise, they retained high fidelity in their annotations (over 84% precision for PubMedDS, and 91% CUI-level accuracy in WikiMed) and our results demonstrate their utility in training semantic type prediction models. We make the source code for our experiments and our two novel datasets available to the community from http://github.com/svjan5/medtype, as a resource for further research on biomedical information extraction.

## Figures and Tables

**Table 1:** Grouping of the 127 semantic types in the UMLS Metathesaurus into 24 semantic groups. The semantic groups were derived from McCray et al. [48] and *is-a* relationships in the Semantic Network. Refer to Section 3.3 for details.

Groups	Semantic Types
Activities & Behaviors	Activity, Behavior, Daily or Recreational Activity, Event, Governmental or Regulatory Activity, Individual Behavior, Machine Activity, Occupational Activity, Social Behavior
Anatomy	Anatomical Structure, Body Location or Region, Body Part, Organ, or Organ Component, Body Space or Junction, Body Substance, Body System, Cell, Cell Component, Embryonic Structure, Fully Formed Anatomical Structure, Tissue
Chemicals & Drugs	Amino Acid, Peptide, or Protein, Antibiotic, Biologically Active Substance, Biomedical or Dental Material, Chemical, Chemical Viewed Functionally, Chemical Viewed Structurally, Element, Ion, or Isotope, Enzyme, Hazardous or Poisonous Substance, Hormone, Immunologic Factor, Indicator, Reagent, or Diagnostic Aid, Inorganic Chemical, Nucleic Acid, Nucleoside, or Nucleotide, Receptor, Vitamin
Concepts & Ideas	Classification, Conceptual Entity, Group Attribute, Idea or Concept, Intellectual Product, Language, Quantitative Concept, Regulation or Law, Spatial Concept, Temporal Concept
Devices	Drug Delivery Device, Medical Device, Research Device
Disease or Syndrome	Disease or Syndrome
Disorders	Acquired Abnormality, Anatomical Abnormality, Cell or Molecular Dysfunction, Congenital Abnormality, Experimental Model of Disease, Injury or Poisoning
Finding	Finding
Functional Concept	Functional Concept
Genes & Molecular Sequences	Amino Acid Sequence, Carbohydrate Sequence, Gene or Genome, Molecular Sequence, Nucleotide Sequence
Living Beings	Age Group, Amphibian, Animal, Archaeon, Bacterium, Bird, Eukaryote, Family Group, Fish, Fungus, Group, Human, Mammal, Organism, Patient or Disabled Group, Plant, Population Group, Professional or Occupational Group, Reptile, Vertebrate, Virus
Mental or Behavioral Dysfunction	Mental or Behavioral Dysfunction
Neoplastic Process	Neoplastic Process
Objects	Geographic Area, Entity, Food, Manufactured Object, Physical Object, Substance
Occupations	Biomedical Occupation or Discipline, Occupation or Discipline
Organic Chemical	Organic Chemical
Organizations	Health Care Related Organization, Organization, Professional Society, Self-help or Relief Organization
Pathologic Function	Pathologic Function
Pharmacologic Substance	Clinical Drug, Pharmacologic Substance
Phenomena	Biologic Function, Environmental Effect of Humans, Human-caused Phenomenon or Process, Laboratory or Test Result, Natural Phenomenon or Process, Phenomenon or Process
Physiology	Cell Function, Clinical Attribute, Genetic Function, Mental Process, Molecular Function, Organ or Tissue Function, Organism Attribute, Organism Function, Physiologic Function
Procedures	Diagnostic Procedure, Educational Activity, Health Care Activity, Laboratory Procedure, Molecular Biology Research Technique, Research Activity, Therapeutic or Preventive Procedure
Qualitative Concept	Qualitative Concept
Sign or Symptom	Sign or Symptom

**Table 2:** Frequencies of semantic types in our evaluation datasets and novel training datasets. Overall, we find that our WikiMed and PubMedDS datasets give diverse coverage across all semantic types.

	*Evaluation datasets*				*Novel datasets*	
Categories	NCBI	Bio CDR	ShARe	MedMentions	WikiMed	PubMedDS
Activities & Behaviors	4	7	1	12,249	554	2,725,161
Anatomy	3	29	4	19,098	14,366	10,688,138
Chemicals & Drugs	0	32,436	1	46,420	26,809	44,476,957
Concepts & Ideas	0	0	1	60,475	2,562	5,274,354
Devices	0	0	0	2,691	483	242,599
Disease or Syndrome	10,760	22,603	5,895	11,709	84,706	9,846,667
Disorders	664	1,853	997	3,575	8,635	1,115,186
Finding	749	2,220	500	15,666	9,285	1,778,023
Functional Concept	0	0	1	23,672	117	48,553
Genes & Molecular Sequences	20	0	0	5,582	446	281,662
Living Beings	0	43	7	31,691	919,694	21,339,662
Mental or Behavioral Dysfunction	293	3,657	410	2,463	19,196	2,353,547
Neoplastic Process	4,022	2,301	323	4,635	16,823	1,476,843
Objects	0	129	2	10,357	421	5,184,355
Occupations	0	0	0	1,443	1,156	654,604
Organic Chemical	0	90,428	1	10,258	17,330	50,248,085
Organizations	0	0	0	2,276	0	298,119
Pathologic Function	143	3,290	2,285	4,121	4,474	1,895,835
Pharmacologic Substance	0	90,872	1	11,935	24,878	50,696,769
Phenomena	4	163	2	7,210	317	1,722,873
Physiology	15	166	3	24,753	2,054	10,674,561
Procedures	5	73	4	37,616	4,008	7,471,434
Qualitative Concept	0	0	7	32,564	106	1,211,747
Sign or Symptom	211	9,844	2,687	1,809	4,212	3,750,734

**Table 3:** Quality assessment of PubMedDS, based on the subset of documents it shares with the NCBI Disease Corpus, Bio CDR, and MedMentions. Precision and recall are calculated with respect to overlap between our automated annotations in PubMedDS and the gold standard annotations in the comparison datasets. We find that although PubMedDS has low coverage, extracted mentions have high precision across the three datasets.

Documents shared with	Precision	Recall
NCBI	86.3	6.5
Bio CDR	75.8	1.3
MedMentions	90.3	5.3

**Table 4:** Details of the medical entity linking datasets used in our experiments; #Unq Con refers to the number of unique CUIs in each dataset. WikiMed is our novel automatically-annotated Wikipedia dataset, and PubMedDS is our novel distantly supervised dataset.

Datasets	#Documents	#Sentences	#Mentions	#Unq Concepts
NCBI	792	7,645	6,817	1,638
Bio CDR	1,500	14,166	28,559	9,149
ShARe	431	27,246	17,809	1,719
MedMentions	4,392	42,602	352,496	34,724
WikiMed	393,618	11,331,321	1,067,083	57,739
PubMedDS	13,197,430	127,670,590	57,943,354	44,881

**Table 5:** Semantic type prediction results, comparing MedType (with and without additional corpora) to our four baselines; we report the area under the precisionrecall curve as our evaluation metric. MT ← X denotes MedType first trained on X dataset then fine-tuned using T. We find that MedType outperforms other methods on 3 out of 4 datasets. Also, pre-training on WikiMed and PubMedDS gives substantial boost in the performance. More details are provided in Section 6.1.

	NCBI	Bio CDR	ShARe	MedMentions
AttentionNER [77]	94.5	89.1	88.7	72.0
DeepType-FC [22]	95.1	82.9	89.3	72.9
DeepType-RNN [22]	92.8	86.9	86.1	74.1
Type-CNN [78]	95.2	88.9	89.8	74.4
MedNER [36]	95.6	90.2	84.4	67.5
MedType (MT)	94.5	90.4	90.5	83.5
MT ← WikiMed	94.9	93.5	93.2	84.0
MT ← PubMedDS	96.8	**97.3**	93.6	86.8
MT ← Both	**97.2**	**97.3**	**95.1**	**87.3**

**Table 6:** For quantifying the impact of semantic type prediction on medical entity linking, we report the F1-score for five medical entity linking methods on multiple datasets. For each method, the first row is its base performance, and the following rows indicate the change in F1-score on incorporating a type-based candidate concepts filtering step. **Bold** indicates the case when MedType performance matches with an oracle. We report the results with the oracle type predictors (fine-grained and coarse-grained) and MedType. Overall, we find that MedType gives performance comparable to an oracle and improves medical entity linking across all settings. Please refer to Section 6.2 for details.

	NCBI		Bio CDR		ShARe		MedMentions	
	Exact	Partial	Exact	Partial	Exact	Partial	Exact	Partial
**MetaMap**	39.6	45.0	54.2	56.3	33.8	34.6	36.7	39.8
Oracle (Fine)	+0.8	+1.0	+0.3	+0.4	+0.5	+0.6	+6.4	+6.9
Oracle (Coarse)	+0.8	+1.0	+0.2	+0.3	+0.5	+0.6	+5.7	+6.1
Type-CNN	+0.7	+0.8	+0.2	+0.3	+0.2	+0.3	+3.6	+3.8
MedType	**+0.8**	**+1.0**	**+0.2**	**+0.3**	+0.3	+0.4	+4.0	+4.3
**cTakes**	39.2	45.9	54.5	57.0	32.3	33.3	16.9	18.3
Oracle (Fine)	+0.3	+0.3	+0.1	+0.2	+0.1	+0.2	+0.2	+0.2
Oracle (Coarse)	+0.3	+0.3	+0.1	+0.2	+0.1	+0.2	+0.2	+0.2
Type-CNN	+0.3	+0.3	+0.1	+0.2	+0.0	+0.1	+0.1	+0.1
MedType	**+0.3**	**+0.3**	**+0.1**	**+0.1**	+0.1	+0.1	**+0.2**	**+0.2**
**MetaMapLite**	35.4	39.4	50.3	51.5	27.1	27.5	32.6	35.2
Oracle (Fine)	+5.9	+5.9	+2.7	+2.8	+4.7	+4.8	+7.2	+7.8
Oracle (Coarse)	+5.9	+5.9	+2.6	+2.7	+4.7	+4.7	+6.0	+6.5
Type-CNN	+5.7	+5.7	+2.3	+2.4	+4.1	+4.1	+3.9	+4.0
MedType	**+5.9**	**+5.9**	+2.5	+2.6	+4.3	+4.4	+4.4	+4.6
**QuickUMLS**	27.0	31.7	36.5	39.1	17.3	19.2	28.7	31.4
Oracle (Fine)	+0.2	+0.6	+5.0	+5.2	+5.2	+5.5	+9.8	+10.7
Oracle (Coarse)	+0.2	+0.6	+4.5	+4.6	+5.1	+5.4	+7.7	+8.5
Type-CNN	+0.0	+0.2	+4.0	+4.1	+4.0	+4.2	+4.9	+5.2
MedType	+0.1	+0.5	+4.3	+4.4	+4.8	+5.0	+5.9	+6.4
**ScispaCy**	43.1	47.5	49.4	53.7	25.4	29.0	37.2	40.6
Oracle (Fine)	+2.2	+4.1	+1.7	+2.6	+3.5	+5.1	+8.2	+9.4
Oracle (Coarse)	+2.2	+4.1	+1.7	+2.5	+3.4	+5.0	+6.8	+7.8
Type-CNN	+1.7	+3.6	+0.5	+1.2	+2.9	+4.0	+3.5	+3.9
MedType	+1.9	+3.8	+1.3	+2.2	+3.1	+4.5	+4.1	+4.6

**Table 7:** Results of *Partial*_*mention*_*id*_*match* evaluation of ScispaCy on all four evaluation datasets. Evaluation is restricted to only predicted samples that overlap with gold annotations, to control for the effects of mention detection errors. The number of samples in this restricted subset of each dataset is given in the column headers.

	NCBI (1,042)	Bio CDR (9,243)	ShARe (6,691)	MedMentions (61,367)
ScispaCy	56.0	60.9	30.9	42.8
Oracle (Fine)	+4.2	+2.7	+5.3	+9.9
Oracle (Coarse)	+4.2	+2.6	+5.3	+8.1
Type-CNN	+3.5	+1.2	+4.2	+4.1
**MedType**	+3.8	+2.2	+4.7	+4.9

**Table 8:** Type-wise analysis of the impact on using MedType with PubMedDS on NCBI, Bio CDR, ShARe, and MedMentions datasets. We report F1-score for each semantic type. MT denotes MedType, ← W and ← P indicate MedType first pre-trained on WikiMed and PubMedDS dataset, and ← B denotes MedType pre-trained on both the datasets. ‘-’ mean that the semantic type was not part of the dataset.

	NCBI				Bio CDR				ShARe				MedMentions			
	MT	← W	← P	← B	MT	← W	← P	← B	MT	← W	← P	← B	MT	← W	← P	← B
Activities & Beh.	-	-	-	-	-	-	-	-	-	-	-	-	71.9	71.7	74.4	**74.9**
Anatomy	-	-	-	-	-	-	-	-	-	-	-	-	81.3	82.7	**86.5**	86
Chemicals & Drugs	-	-	-	-	83	83	91.5	**91.8**	-	-	-	-	77.8	78.1	**82.2**	**82.2**
Concepts & Ideas	-	-	-	-	-	-	-	-	-	-	-	-	80.5	81.2	**82.8**	**82.8**
Devices	-	-	-	-	-	-	-	-	-	-	-	-	52.2	46.4	**55.5**	54.1
Disease or Syn.	94.5	95.5	97.2	**97.6**	87.8	90.5	93.2	**93.7**	84.6	91.3	92.3	**92.8**	79	81	84.4	**84.9**
Disorders	58.9	68.7	69	**69.2**	82.4	79.4	**85.8**	85.7	50.7	78	79.9	**82.1**	62.1	64.4	67.9	**68.6**
Finding	0	45	46.8	**51.2**	59.6	77.1	86.1	**87.6**	47.5	79.5	82.5	**83.3**	54.8	57.5	58.5	**59.8**
Functional Concept	-	-	-	-	-	-	-	-	-	-	-	-	76.7	76.4	77.2	**77.4**
Genes & Mol. Seq.	-	-	-	-	-	-	-	-	-	-	-	-	67.8	67	**72**	**72**
Living Beings	-	-	-	-	0	0	**57.1**	40	-	-	-	-	88.1	88.6	90.1	**90.1**
Mental/Beh. Dys.	17.4	81.1	**83.3**	**83.3**	58.8	90.1	92.6	**92.9**	48.4	83.2	78.8	**85.4**	76.7	79	80.7	**82.2**
Neoplastic Process	91.7	93.1	**94.2**	92.7	90.9	90.8	**94.6**	92.2	71.5	89.2	90.9	**91.4**	85.6	86	87.4	**88.1**
Objects	-	-	-	-	0	20.8	**46.4**	29.2	-	-	-	-	72.3	71.6	75.7	**76.1**
Occupations	-	-	-	-	-	-	-	-	-	-	-	-	46.7	47.1	**58.4**	55.5
Organic Chemical	-	-	-	-	91.9	91.3	**94.3**	94.1	-	-	-	-	71.9	73.6	**80.6**	80.2
Organizations	-	-	-	-	-	-	-	-	-	-	-	-	73	74	75.6	**77.3**
Pathologic Function	0	76.2	**82.4**	80	59.6	86.2	90.2	**91**	74.6	85.1	85.9	**86.5**	65.6	69.9	70.1	**72.7**
Pharm. Substance	-	-	-	-	92	91.8	**93.3**	93.1	-	-	-	-	63.6	64.3	**70.8**	70.3
Phenomena	-	-	-	-	33.3	74.3	**93.8**	92.3	-	-	-	-	51.1	54.3	**61.5**	60.7
Physiology	-	-	-	-	0	60.8	**63.7**	60.8	-	-	-	-	72.7	74.6	77.3	**77.8**
Procedures	-	-	-	-	0	0	44.4	**53.3**	-	-	-	-	77.1	78.3	**80.3**	80.2
Qualitative Concept	-	-	-	-	-	-	-	-	-	-	-	-	82.8	83.5	84.1	**84.4**
Sign or Symptom	0	81.8	**83.3**	**83.3**	46.4	89.5	89.9	**91.7**	80.6	92.8	**94.7**	94.4	72.1	75.4	75.1	**78.9**

**Table 9:** Most frequent confusions in semantic type predictions on the MedMentions validation set, using MedType pretrained on WikiMed and PubMedDS.

Target Semantic Type	Top Confused Semantic Types
Devices	Concepts & Ideas, Objects, Procedures,
Disorders	Disease or Syndrome, Finding
Finding	Concept & Ideas, Physiology, Functional Concept
Functional Concept	Procedures, Concepts & Ideas
Genes & Mol. Sequences	Chemicals & Drugs
Mental and Behavioral Dys.	Disease or Syndrome, Finding
Objects	Concepts & Ideas, Chemicals & Drugs
Occupations	Procedures, Concepts & Ideas, Functional Concepts
Organic Chemicals	Chemicals & Drugs, Pharmacological Substances
Organizations	Concepts & Ideas, Procedures, Living Beings
Pathologic Functions	Disease or Syndrome, Finding, Functional Concepts
Pharmacological Substance	Chemical & Drugs, Organic Chemicals

## Footnotes

1 Counts taken from UMLS 2019AB release.

2 https://cwiki.apache.org/confluence/display/cTAKES/cTAKES+4.0+-+LVG.

3 https://tac.nist.gov/2013/KBP/.

4 https://github.com/wikilinks/neleval.

5 We note that WikiMed and PubMedDS should not, however, be used to train biomedical mention detection (NER) systems, as the automated annotation process emphasized precision over recall and many potentially valid concept mentions were not included due to missing links (wiki) or MeSH headers (PubMedDS).
